# Number of Regularly Prescribed Drugs and Intrapatient Tacrolimus Trough Levels Variability in Stable Kidney Transplant Recipients

**DOI:** 10.3390/jcm9061926

**Published:** 2020-06-19

**Authors:** Piotr Giza, Rafał Ficek, Tomasz Dwulit, Jerzy Chudek, Iwona Woźniak, Andrzej Więcek, Aureliusz Kolonko

**Affiliations:** 1 Department of Nephrology, Transplantation and Internal Medicine, Medical University of Silesia, Francuska 20/24, 40-027 Katowice, Poland; piotr-giza@gmail.com (P.G.); rficek@me.com (R.F.); tomek.dwulit@gmail.com (T.D.); awiecek@sum.edu.pl (A.W.); 2 Department of Internal Medicine and Oncological Chemotherapy, Medical University of Silesia, Reymonta 8, 40-027 Katowice, Poland; chj@poczta.fm; 3 University Hospital, Medical University of Silesia, Francuska 20/24, 40-027 Katowice, Poland; iwona.w1@o2.pl

**Keywords:** chronic kidney disease, coefficient of variation, co-medication, drug interactions, number of medications, extended-release tacrolimus, tacrolimus formulation

## Abstract

High intra-patient variability (IPV) of tacrolimus levels is associated with poor long-term outcome after transplantation. We aimed to evaluate whether the number of regularly prescribed medications is associated with the tacrolimus IPV. We have studied 152 kidney transplant recipients (KTRs) with mean post-transplant time of 6.0 ± 3.1 years. The coefficient of variation (CV) as a measure of IPV was calculated in each individual patient. Data concerning the type and number of currently prescribed medications were collected. The participants were divided into four groups, based on the number of regularly prescribed drugs (≤3, 4–6, 7–9, ≥10 drugs, respectively). There was an increasing trend for median CV, proportional to the increasing number of medications [group 1: 0.11 (interquartile range, 0.08–0.14), group 2: 0.14 (0.01–0.17), group 3: 0.17 (0.14–0.23), group 4: 0.17 (0.15–0.30); *p* value for trend = 0.001]. Stepwise backward multivariate regression analysis revealed that the number of medications [partial correlation coefficient (*r*_partial_) = 0.503, *p* < 0.001] independently influenced the tacrolimus IPV. Concomitant steroid or diuretics use increased IPV only in Advagraf-treated KTRs, whereas proton-pump inhibitor or statin use increased IPV in the Prograf group but not in the Advagraf group. A large number of concomitant medications significantly increases the tacrolimus IPV in stable KTRs.

## 1. Introduction

Kidney transplantation is an optimal method of treatment in patients with end-stage chronic kidney disease (CKD) [1]. Nowadays, tacrolimus, mycophenolate, and steroids are key immunosuppressive agents in kidney transplantation [2]. Importantly, long-term kidney transplant success is related to the adequate net dose of immunosuppressive drugs, as any acute rejection episode markedly worsens the distant kidney graft outcome [3,4]. Both inadequate low tacrolimus trough levels within the first post-transplant year and high intra-patient variability (IPV) of tacrolimus blood levels are associated with subsequent poor long-term outcome after kidney transplantation [5,6]. Additionally, high tacrolimus IPV is associated with accelerated progression of chronic histologic lesions in kidney transplant recipients (KTRs) [7].

As early as in 1976, the term “iatrogenic disease” was introduced for diseases caused by prescribed therapy [8]. Adverse effects of drugs are more common in older and seriously ill patients. Polypharmacy increases the risk, and drug interactions are often the cause of cumulative pharmacologic consequences and individual variability. It is well known that CKD patients are characterized by high comorbidity rate, as they are frequently suffering from cardiovascular disease, diabetes mellitus, metabolic disturbances, and other clinical conditions [9]. As a consequence, an average medication burden in these patients is high, especially in the early post-transplant period [10,11]. This effect is further magnified by the increasing percentage of kidney transplantations performed in patients older than 65 years [12]. Thus, both increasing age and a high prevalence of neurological or psychiatric comorbidity may result in serious cognitive impairment and, in consequence, deteriorate adherence [13]. Moreover, tacrolimus was shown to affect the cognitive functioning of renal transplant recipients by itself [14]. All the aforementioned evidence suggests that high pill burden may reduce the drug adherence and increase the tacrolimus IPV in KTRs. Introduction of once-daily tacrolimus formulation into the clinical practice aimed to improve the drug adherence and the early post-conversion reports were very promising [15,16]. However, the relationship between the number of regularly prescribed drugs and tacrolimus IPV has not been investigated yet. Therefore, we performed this cross-sectional study in a cohort of stable KTRs in order to determine whether the number of regularly prescribed medications is associated with the variability of tacrolimus blood trough levels.

## 2. Materials and Methods

### 2.1. Study Group

One hundred fifty-two stable adult kidney graft recipients, who were at least 12 months after successful kidney transplantation, were enrolled into this cross-sectional study. All patients were treated with Prograf^®^ or Advagraf^®^ and regularly attended the ambulatory clinic, usually every 3 months. Patients with viral hepatitis or diagnosed liver cirrhosis were excluded from this study. The Bioethics Committee of the Medical University of Silesia granted permission for completing the survey (approval number KNW/0022/KB/167/15) and for performing this analysis based on anonymous data. Informed consent was not necessary, as the data analysis does not meet the criteria of medical experiment.

Based on the prospectively operated center database, we identified those patients in whom there were no changes in the tacrolimus formulation and dosing throughout 2 consecutive years. To calculate the tacrolimus IPV, we analyzed nine consecutive measurements of drug blood trough concentrations. Additionally, we reviewed the ambulatory outpatient documentation to eliminate biased measurements, when blood was withdrawn after the ingestion of tacrolimus morning dose or current patient’s complaints included preceding diarrhea. Those biased measurements were excluded from the final analysis. Blood samples for measurement of tacrolimus through concentrations were routinely withdrawn 12 h after the evening dose in patients receiving Prograf or 24 h after the morning dose in patients treated with Advagraf. Tacrolimus blood concentrations were assessed using the microparticle enzyme immunoassay (Abbott Laboratories, Abbott Park, IL, USA). The mean tacrolimus blood concentration, standard deviation, and coefficient of variation (CV) were calculated in each patient.

Data concerning the type (generic name) and number of current medications, weekly pill burden, and dosing frequency were collected by interview survey, performed during routine ambulatory visit. In diabetic patients, insulin formulations were not counted into the number of oral medications taken regularly. In the case of fixed-dose medications consisting of more than one active substance, they were counted separately. Additionally, we present the proportion of renally excreted drugs, taking >50% of urinary elimination as a cut-off value. During the whole 24-month study period, the number of regularly prescribed medications did not change by more than one. For the initial comparison, all participants were divided into four subgroups, according to the number of currently prescribed medications. We also performed another analysis, comparing patients treated with two different tacrolimus formulations: twice-daily Prograf and once-daily Advagraf. For those two groups, we also analyzed the potential influence of different tacrolimus formulation on the association between particular comedication drug classes and the tacrolimus IPV.

Comorbidity was analyzed based on the medical records, and Charlson Comorbidity Index (CCI) was calculated.

### 2.2. Data and Statistical Analysis

Nutritional status was scored according to World Health Organization criteria, based on anthropometric measurements performed during a study outpatient visit [overweight: body mass index (BMI) 25.0–29.9 kg/m^2^, obese: BMI ≥ 30.0 kg/m^2^)].

Statistical analyses were performed using the STATISTICA 13.3 PL for Windows software package (Tibco Inc., Palo Alto, CA, USA) and MedCalc (MedCalc Software, Mariakerke, Belgium). Values are presented as means and 95% confidence intervals or frequencies. Variables with skewed distribution were presented as medians and interquartile range (IQR). The initial comparison was performed for groups defined by the number of medications prescribed (subgroup 1: ≤3 drugs, subgroup 2: 4–6 drugs, subgroup 3: 7–9 drugs, subgroup 4: ≥10 drugs). The subgroups were compared using the χ^2^ test (qualitative variables) and analysis of variance or Kruskal–Wallis test (quantitative variables). Correlation coefficients were calculated according to Pearson. The next comparison was performed for two groups, according to the different tacrolimus formulations (twice daily or once daily), using Mann–Whitney U test. Multivariate regression analyses (stepwise backward model) were performed for the extent of tacrolimus IPV, quantified with CV value, including age, gender, BMI, estimated glomerular filtration rate (eGFR), CCI, the tacrolimus formulation (twice daily or once daily), and the number of regularly prescribed medications and daily dosing frequency. In all statistical tests, *p* values less than 0.05 were considered as statistically significant.

## 3. Results

The study cohort consisted of 79 men and 73 women, including 70 patients treated with twice-daily (Prograf) and 82 patients treated with once-daily (Advagraf) tacrolimus formulation. The mean time post-kidney transplantation was 6.0 ± 3.1 years (range, 1.5–17.1 years). The average number of medications taken regularly by study subjects was 6.2 ± 2.5 (range, 2–15). In the whole study cohort, there were 23 patients with maximum 3 different medications (subgroup 1), 65 patients with 4 to 6 medications (subgroup 2), 50 patients with 7 to 9 medications (subgroup 3), and 14 patients receiving at least 10 different medications (subgroup 4). The clinical characteristics of the study cohort are given in Table 1. Age, gender distribution, time after transplantation, and dialysis vintage before transplantation did not differ significantly between the analyzed subgroups. There was a trend for lower eGFR values along those medication groups. There was also a trend for higher BMI values as well as greater occurrence of hypertension, diabetes mellitus, and cardiovascular and cerebrovascular episodes along the subgroups with increasing number of medications (Table 1). As a consequence, there was also an increasing trend for a higher CCI score along all the four analyzed subgroups. As expected, the mean total weekly pill burden and median dosing frequency per day were proportional to the number of medication.

### 3.1. Tacrolimus IPV and the Number of Regularly Prescribed Medications

**Figure 1:**
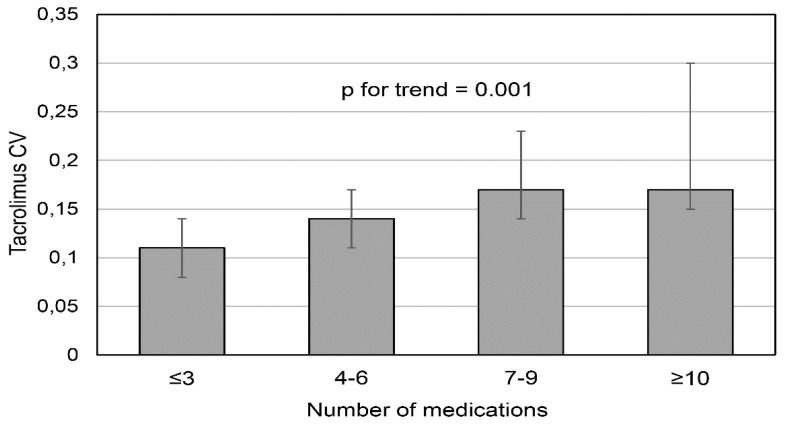
The variability of tacrolimus blood trough levels measured by coefficient of variation (CV) in the subsequent groups based on the increasing number of medications.

**Figure 2:**
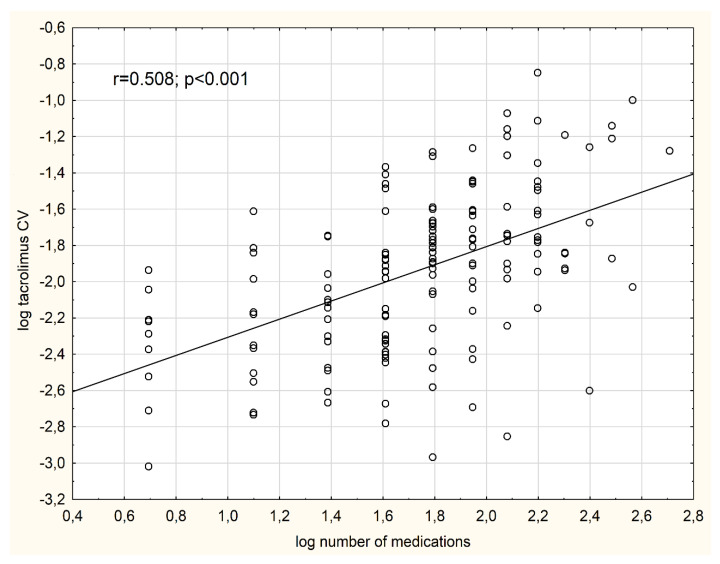
The association between the logarithmized values of tacrolimus CV and the logarithmized number of medications.

In the whole study cohort, the median CV was 0.15 (IQR, 0.11–0.19). The tacrolimus IPV differed significantly between the analyzed subgroups. There was an increasing trend for median CV, proportional to the increasing number of medications [subgroup 1: 0.11 (IQR, 0.08–0.14), subgroup 2: 0.14 (0.1–0.17), subgroup 3: 0.17 (0.14–0.23), subgroup 4: 0.17 (0.15–0.30), *p* value for trend = 0.001] (Figure 1). There was a significant association between the logarithmized number of regularly prescribed medications and log CV (*r* = 0.508, *p* < 0.001) (Figure 2). Additionally, there were significant positive correlations between log CV and BMI (*r* = 0.255, *p* = 0.001), logarithmized frequency of drug dosing per day (*r* = 0.307, *p* < 0.001), a total weekly pill burden (*r* = 0.494, *p* < 0.001), and an inverse correlation between log CV and eGFR (*r* = −0.220, *p* < 0.01). Of note, there was no significant correlation between log CV and log CCI (*r* = 0.107, *p* = 0.19).

Stepwise backward multivariate regression analysis revealed that the number of medications (partial correlation coefficient (*r*_partial_) = 0.503, *p* < 0.001), age [(*r*_partial_ = −0.150, *p* = 0.07] and eGFR [(*r*_partial_ = −0.140, *p* = 0.09] independently influenced the tacrolimus IPV (*R^2^* = 0.30), measured by CV.

### 3.2. Tacrolimus IPV for Two Different Tacrolimus Formulations

There were no significant differences with regard to age, gender distribution, time after transplantation, dialysis vintage, and eGFR. Patients treated with Advagraf were characterized by significantly higher BMI (Table 2). The prevalence of hypertension and CVD comorbidity was similar; however, there was borderline greater occurrence of diabetes (28.1% vs. 15.7%, *p* = 0.07) and borderline greater CCI score in the Advagraf group (Table 2). As expected, the median number of tacrolimus pills per week was significantly lower in the Advagraf group [14 (7–14) vs. 21 (14–28) in the Prograf group, *p* < 0.001]. The mean total weekly pill burden and median dosing frequency per day were similar, whereas the median number of medications was slightly greater in the Advagraf group (Table 2). Of note, the presence of diabetes resulted in significantly greater number of medications [median, 7 (6–9) vs. 6 (4–7) in non-diabetic patients, *p* < 0.001], and mean total weekly pill burden [80 (70–91) vs. 64 (59–68) tablets in non-diabetic patients, *p* < 0.001].

There was no significant difference in the tacrolimus IPV in patients treated with Prograf or Advagraf (Figure 2). Median CV [0.15 (0.1–0.20) in the Prograf group vs. 0.15 (0.11–0.19) in the Advagraf group] was comparable. In both analyzed tacrolimus formulation subgroups, we observed similar associations of log CV with BMI, log number of medications, and log dosing frequency. There was positive correlation between log CV and the time after transplantation in the Prograf (*r* = 0.285, *p* < 0.05) but not in the Advagraf group (*r* = −0.06, not statistically significant). Additionally, CV negatively correlated with eGFR in the Prograf group (*r* = −0.252, *p* < 0.05) but not in the Advagraf group (*r* = −0.188, not statistically significant).

### 3.3. Co-Medication and Tacrolimus IPV

In the whole analyzed cohort, 65 patients were treated with steroids, 41 regularly received oral anticoagulants or antiplatelet drugs; 33, proton pump inhibitors (PPIs); 31, diuretics; 28, statins; 13, oral hypoglycemic drugs; and 8 patients received fibrates. The percentage of renally excreted drugs did not differ between study subgroups (Table 1). Patients treated with steroids had significantly higher tacrolimus IPV [median CV, 0.17 (IQR, 0.13–0.23) vs. 0.14 (0.09–0.17) in group without steroids; *p* < 0.001]. Similarly, there were significantly higher median CV values in patients treated with PPIs [0.18 (0.14–0.24) vs. 0.14 (0.01–0.17), respectively; *p* < 0.001], diuretics [0.17 (0.15–0.28) vs. 0.14 (0.01–0.18), respectively; *p* < 0.01] and statins [0.17 (0.13–0.23) vs. 0.15 (0.1–0.18), respectively; *p* < 0.05].

**Figure 3:**
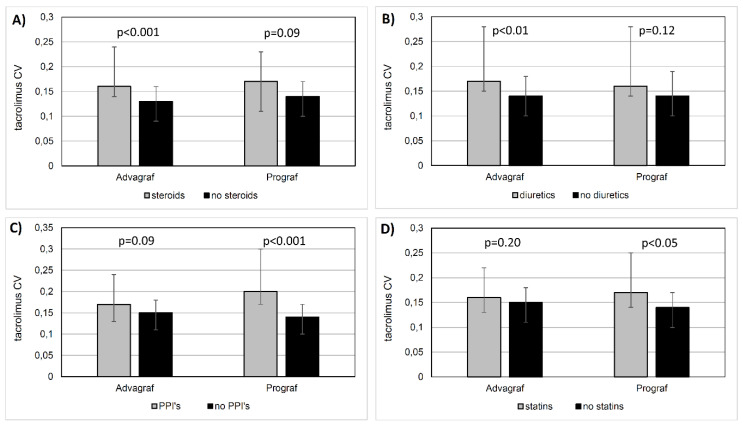
The comparison of tacrolimus coefficient of variation (CV) values in patients treated with once-daily or twice-daily tacrolimus formulation as depends on different classes on concomitant medication: (**A**) steroids, (**B**) diuretics, (**C**) proton pump inhibitors, and (**D**) statins.

Importantly, the potential influence of particular concomitantly received medication on the tacrolimus IPV was determined by the tacrolimus formulation. The statistically significant difference in median CV conditioned by the concomitant steroid use was present only in the Advagraf group [steroids: 0.16 (0.14–0.24) vs. no steroids: 0.13 (0.09–0.16), *p* < 0.001] but not in the Prograf group [steroids: 0.17 (0.11–0.23) vs. no steroids: 0.14 (0.1–0.17), *p* = 0.09] (Figure 3A). Correspondingly, the effect of diuretics use on median CV was seen in the Advagraf group [diuretics: 0.17 (0.15–0.28) vs. no diuretics: 0.14 (0.1–0.18), *p* < 0.01] but not in the Prograf group [diuretics: 0.16 (0.14–0.28) vs. no diuretics: 0.14 (0.1–0.19), *p* = 0.12] (Figure 3B). In contrast, the statistically significant difference in median CV conditioned by the concomitant PPIs use was present in the Prograf group [PPIs: 0.20 (0.17–0.30) vs. no PPIs: 0.14 (0.1–0.17), *p* < 0.001] but not in the Advagraf group [PPIs: 0.17 (0.13–0.24) vs. no PPIs: 0.15 (0.11–0.18), *p* = 0.09] (Figure 3C). Similarly, the effect of statins on median CV was seen in the Prograf group [statins: 0.17 (0.16–0.25) vs. no statins: 0.14 (0.1–0.17), *p* < 0.05] but not in the Advagraf group [statins: 0.16 (0.13–0.22) vs. no statins: 0.15 (0.11–0.18); *p* = 0.20] (Figure 3D).

## 4. Discussion

The most important finding of the study is the association between the number of regularly prescribed drugs and the tacrolimus IPV in stable KTRs, which was independent of the comorbidity. Unexpectedly, the tacrolimus IPV did not differ between both analyzed tacrolimus formulations (Prograf or Advagraf). Instead, the tacrolimus IPV in KTRs receiving Prograf but not Advagraf was increased by the worse kidney graft excretory function and longer post-transplantation time. Lastly, we described the opposing effect of different tacrolimus formulations, when analyzing the influence of several concomitantly received classes of medications, including steroids, diuretics, PPIs, and statins, on the tacrolimus IPV.

During the long-term follow-up period after kidney transplantation, despite the careful therapeutic drug monitoring, the intra-patient blood trough levels of tacrolimus may differ substantially. Except the influence of food ingestion timing and occasional diarrhea, the main determinants of drug concentration instability are inadequate adherence and multiple drug-drug interactions, including steroids, azole antifungals, rifampin, several antiepileptic drugs, and others [17]. As the medication burden in KTRs is high, as compared with other chronic illness [10], it could suggest a high probability of medication errors in this particular population, caused by the complexity of drug regimens, variability among patient medication-management skills, and frequent drug-drug interactions [18]. Moreover, the number of drugs dispensed was the strongest predictor of the use of potentially inappropriate medications in older adults [19]. On the other hand, the pill burden negatively influenced the quality of life in patients after kidney transplantation [20]. In fact, in a cross-sectional study in 408 KTRs, the only variable confirmed by multivariate analysis as an independent predictor of high calcineurin inhibitors variability (>30%) was the presence of discrepancies in drug regimen, identified by a clinical pharmacist interview [21]. In our present study, the high number of oral medications was the only significant risk factor, which independently increased the tacrolimus IPV calculated for a 24-month period in stable KTRs. Additionally, negative influence of the patient’s age and eGFR was identified as of borderline significance, which is in line with previous findings, as both those factors are associated with cognitive dysfunction [13]. Notably, the co-morbidity, quantified with the use of CCI, was not confirmed as an independent factor in multivariate or linear regression analysis. Taking into account that the number of medications in the first post-transplant year is much higher than in the later observation period [10], the early risk of substantially higher tacrolimus variability and, consequently, worse long-term kidney graft outcome [6,22], is significant.

After the introduction of extended-release tacrolimus formulation (Advagraf) into the clinical practice, the greater tacrolimus medication adherence and stability of blood levels were anticipated [23]. Early studies reported a significant reduction of tacrolimus IPV after conversion from twice-daily to once-daily formulation [15,24]. However, there were some methodological inaccuracies, which may influence the described findings [25]. Furthermore, the tacrolimus IPV was decreased, when CV was calculated based on the area under the curve values but not on the trough levels [15]. The similar pre-conversion and post-conversion tacrolimus IPV was also shown by Shuker et al. [25]. In our non-conversion study, the tacrolimus IPV in the two subgroups steadily treated with once- or twice-daily tacrolimus formulation was comparable. However, it was negatively influenced, that is, increased, by the worse kidney graft excretory function and longer post-transplantation time only in patients treated with Prograf. The latter finding was previously described by other authors [21]. Importantly, we also described the substantial, tacrolimus formulation dependent differences in the assumed influence of several concomitantly received classes of medications, including steroids, diuretics, PPIs, and statins, on the tacrolimus IPV. Such a differences may result from the unlike effects of both tacrolimus formulations on the effective renal plasma flow, renal blood flow, eGFR and several hormones, including prostaglandins, endothelin, and renin [26]. Moreover, the standard timing of ingestion of particular medication classes may play a role, as the sole doses of steroids and diuretics are usually administered in the morning, and those two classes were shown to increase the drug variability in the Advagraf group. On contrast, both PPIs and statins, usually ingested in the evening hours (for PPIs, this is a specific, center-recommended timing to avoid drug interactions), increased the tacrolimus IPV in Prograf-treated patients. To our knowledge, such relationships have not been previously described. However, they should be taken into consideration, as in the case of a patient’s particular interfering co-medication, the minimization of maintenance doses when using the tacrolimus formulation, which is more prone to high IPV, may increase the risk of underimmunosuppression.

One of the study limitations, except its retrospective nature, is the method of tacrolimus level assessment—microparticle enzyme immunoassay. That is considered inferior to most specific liquid chromatography with mass spectrometric detection, used only by selected transplant centers [17]. Nevertheless, our study had a non-conversion design, and all participants were continuously treated with the same tacrolimus formulation. Another limitation is the fact that the accuracy of recommended time distance between the last tacrolimus dose ingested and the next-day blood withdrawal for trough level measurement was not controlled, as the study was based on outpatient clinic visits. However, we eliminate the obviously biased results, verified by the transplant physician during the appointment. Additionally, we did not perform any accompanying adherence assessments. Finally, we were not able to assess the potential effect of some medication classes with known influence on the tacrolimus IPV because of their incidental use within our study cohort. On the other hand, we carefully selected the study population to eliminate the bias caused by any tacrolimus formulation changes (i.e., the temporary use of generic substitutions, which may negatively affect drug adherence and tacrolimus IPV). We also based our analysis on the nine consecutive trough levels, which probably let us estimate the real tacrolimus variability more reliably than using the prior minimum three-point pattern.

## 5. Conclusions

In summary, the present study reported the important independent association between the number of regularly prescribed drugs and tacrolimus IPV in stable KTRs. Tacrolimus IPV did not differ between both analyzed tacrolimus formulations (Prograf or Advagraf). Of importance, we also described the substantial, tacrolimus formulation dependent differences in the assumed influence of several concomitantly received classes of medications, including steroids, diuretics, PPIs, and statins, on the tacrolimus IPV. This should be kept in mind during the choice of the tacrolimus treatment strategy, as in the case of patient’s interfering co-medication, the minimization of maintenance doses when using the tacrolimus formulation, which is more prone to high IPV, should be avoided.

## Figures and Tables

**Table 1:** The clinical characteristics of patients divided into the study subgroups according to the number of regularly prescribed medications.

	Study Subgroups Divided According to the Number of Medications				*p*
	Subgroup 1	Subgroup 2	Subgroup 3	Subgroup 4	
	≤3 (*N* = 23)	4–6 (*N* = 65)	7–9 (*N* = 50)	≥10 (*N* = 14)	
Age (years)	47.7 (42.9–52.6)	47.7 (44.3–51.1)	51.1 (47.7–54.5)	55.4 (50.6–60.2)	0.13
Gender (M/F)	11/12	39/26	23/27	6/8	0.39 ***
BMI (kg/m^2^)	25.8 (23.8–27.8)	26.0 (25.1–26.9)	28.8 (27.4–30.3)	31.4 (28.8–34.0)	<0.001
Dialysis vintage (months) *	31 (14–42)	29 (18–48)	30 (23–40)	24 (10–44)	0.96 **
Time from transplantation (years)	6.0 (4.6–7.4)	6.3 (5.4–7.1)	5.6 (4.8–6.5)	6.2 (4.7–7.8)	0.74
eGFR (mL/min/1.73m^2^)	70.6 (63.8–77.3)	60.8 (55.2–66.4)	55.5 (49.7–61.4)	49.3 (37.5–61.1)	<0.01
Hypertension (*n* (%))	7 (30.4)	51 (78.5)	49 (98)	12 (85.7)	<0.001
CVD (*n* (%))	2 (9)	6 (9)	8 (16)	5 (36)	0.02 for trend ***
Diabetes (*n* (%))	2 (9)	10 (15)	15 (30)	7 (50)	<0.001 for trend ***
CCI *	3 (2–4)	3 (2–5)	4 (3–5)	5 (3–6)	<0.01 **
Number of medications *	3 (2–3)	5 (5–6)	8 (7–9)	11 (10–12)	<0.001 **
Total weekly pill burden	40 (33–47)	57 (54–61)	81 (77–85)	111 (96–126)	<0.001
Dosing frequency per day *	2 (2–2)	2 (2–3)	3 (2–4)	5 (4–5)	<0.001 **
Renally excreted drugs * (%)	58.3 (50.0–66.7)	50.0 (40.0–66.7)	55.6 (50.0–71.4)	59.2 (50.0–63.6)	0.95 **

Data presented as means and 95% confidence interval, or frequencies, except * median value and interquartile range. Statistics: ANOVA, except ** Kruskal–Wallis test or *** χ2 test. BMI, body mass index; eGFR, estimated glomerular filtration rate calculated according to MDRD formula; CVD, cardio- or cerebrovascular disease; CCI, Charlson Comorbidity index.

**Table 2:** Clinical characteristics of patients divided into the study groups according to the twice daily or once daily prescribed tacrolimus formulation.

	Study Groups According to the Tacrolimus Formulation		*p*
	Prograf (*N* = 70)	Advagraf (*N* = 82)	
Age (years)	49.2 (46.1–52.4)	49.8 (47.1–52.4)	0.78
Gender (M/F)	36/34	43/39	0.90 ***
BMI (kg/m^2^)	26.3 (25.4–27.2)	28.3 (27.2–29.5)	<0.01
Dialysis vintage (months) *	34 (19–48)	25 (15–38)	0.55 **
Time after transplantation (years)	6.2 (5.4–7.0)	5.9 (5.2–6.5)	0.52
eGFR (mL/min/1.73m^2^)	62.5 (57.2–67.7)	56.9 (52.3–61.4)	0.11
Hypertension (*n* (%))	55 (78.6)	64 (78.1)	0.94
CVD (*n* (%))	11 (15.7)	10 (12.2)	0.53 ***
Diabetes (*n* (%))	11 (15.7)	23 (28.1)	0.07 ***
CCI *	3 (2–4)	4 (3–5)	0.06 **
Number of medications *	6 (4–7)	6 (5–8)	<0.05 **
Total weekly pill burden	69.4 (63.7–75.0)	65.6 (59.6–71.5)	0.36
Dosing frequency per day *	3 (2–3)	3 (2–3)	0.37 **

Data presented as means and 95% confidence interval, or frequencies, except * median value and interquartile range. Statistics: Student *t*-test, except ** U Mann–Whitney test or *** χ2 test. BMI, body mass index; eGFR, estimated glomerular filtration rate calculated according to MDRD formula; CVD, cardio- or cerebrovascular disease; CCI, Charlson Comorbidity index.
